# Upcycling of Agro-Waste: Research on Performance of a Novel Super-Hygroscopic Material Prepared by Exploiting the Porous Structure of Steam-Exploded Modified Corn Stalk Pith

**DOI:** 10.3390/polym17131779

**Published:** 2025-06-27

**Authors:** Nan Wang, Chuntao Xia, Tingting Liu, Dawei Wang

**Affiliations:** 1 School of Food Science and Engineering, Jilin Agricultural University, Changchun 130118, China; 18943920137@163.com (N.W.); 15843642571@163.com (C.X.); 2 Key Laboratory of Technological Innovations for Grain Deep-Processing and High-Effeciency Utilization of By-Products of Jilin Province, Changchun 130118, China; 3 Engineering Research Center of Grain Deep-Processing and High-Effeciency Utilization of Jilin Province, Changchun 130118, China

**Keywords:** agricultural waste utilization, corn stalk pith, steam explosion modification, porous structure, moisture adsorption, sorption model

## Abstract

Herein, a novel super-hygroscopic material, steam-exploded modified corn stalk pith (SE-CSP), was developed from corn stalk pith (CSP) via the steam explosion (SE) method, and its hygroscopic properties and mechanisms were evaluated. The results confirmed that SE effectively removed lignin and hemicellulose, disrupted the thin cell walls of natural CSP, and formed an aligned porous structure with capillary channels. SE changed the bonding distribution and surface morphology, and enhanced the crystallinity and thermal stability of CSP. The equilibrium hygroscopic percentage of SE-CSP (62.50%) was higher than that of CSP (44.01%) at 25 °C and 80% relative humidity (RH), indicating significantly greater hygroscopicity. The hygroscopic process of SE-CSP followed a Type III isotherm and fitted the Guggenheim–Anderson–de Boer (GAB), Peleg, and pseudo-first-order kinetic models. This process exhibited multi-layer adsorption with enthalpy-driven, exothermic behavior, primarily through physical adsorption involving hydrogen bonds and van der Waals forces. This work offered a new approach for advancing sorption dehumidification technology.

## 1. Introduction

Air humidity is a critical environmental factor that influences multiple facets of daily life [1], from electronics and pharmaceuticals to food processing and storage, as well as cultural relic protection [2,3,4]. The most commonly employed dehumidification methods are mechanical and adsorption techniques [5,6]. Mechanical dehumidification mainly involves cooling air to a temperature below the dew point, enabling the removal of water vapor from the moist air via condensation, which results in significant energy consumption and raises environmental concerns [7]. In comparison, adsorption dehumidification represents an eco-friendly approach that efficiently leverages the properties of adsorbents, demonstrating significant potential in the integrated development of energy and environmental sectors [8]. Presently, the most commonly utilized hygroscopic materials encompass silica gel, lithium chloride, calcium chloride, and molecular sieves [9,10,11]. These conventional hygroscopic materials, however, have limitations. For example, ceramic hygroscopic materials, such as silica gel, have poor adsorption and desorption kinetic performance, low adsorption capacities, and high regeneration temperatures [12]. Salt-based hygroscopic materials, such as lithium chloride, are prone to deliquescence and can potentially cause corrosion [13]. Thus, numerous researchers have made great efforts toward developing novel hygroscopic materials with low regeneration temperatures and enhanced hygroscopic properties that are environmentally safe.

As China’s grain production has steadily increased, the volume of agricultural waste has correspondingly risen. Recent statistics indicate that China generates 900 million tons of crop straw each year, and corn straw constitutes 32% of it [14]. Despite the government’s proactive measures to promote the efficient utilization of agricultural waste, several persistent challenges remain, including underutilization, high processing costs, and low-value-added products [15]. Therefore, enhancing the efficient use and resource recovery of crop straw is crucial for the development of agricultural industrialization. Corn straw is mainly composed of the corn straw cortex and the corn straw pith (CSP) [16]. According to previous research findings, CSP contains abundant biopolymers such as cellulose, hemicellulose, and lignin, and possesses a naturally spongy porous structure that facilitates liquid flow and penetration [17]. Furthermore, these polymers possess a variety of functional groups, including hydroxyl, phenolic hydroxyl, and carboxyl groups, which exhibit significant adsorption properties for methylene blue, oil substances, water molecules, and heavy metal ions [14,18]. These properties make CSP a promising resource for porous adsorbents, water-retaining materials, activated carbon production, and micro- and nanocellulose feedstock [16,19,20]. Therefore, we speculated that CSP could be used to develop a novel hygroscopic material for effectively controlling the humidity in the air. However, the inherent dense structure of CSP necessitates an appropriate treatment to render it moderately loose and expanded, thereby achieving better adsorption performance [21]. Steam explosion (SE), an emerging physical modification technology, uses rapid steam pressure release at high temperatures and pressures to alter the properties of materials [22]. It has the characteristics of short processing time, high efficiency, and environmental friendliness. Recently, SE has been widely employed for processing crop residues and lignocellulosic resources. Studies have shown that SE can mechanically disrupt the lignocellulosic matrix and remove hemicelluloses, thereby loosening the material structure and enhancing its surface area and porosity [23,24].

Therefore, in this study, we have developed a novel super-hygroscopic material (SE-CSP) using SE-modification technology based on natural CSP. Traditional super-hygroscopic materials are synthetic and petroleum-based products, primarily consisting of polyacrylates, and are non-renewable [25]. Depending on their types, the hygroscopic capacity of these petroleum-based super-hygroscopic polymers (SHPs) can reach as high as 500–1000 g/g [26]. Nevertheless, the production of SHPs involves substantial expenses, high embodied carbon, and poses environmental harm, all of which conflict with the tenets of sustainability. Thus, researchers have been focusing on utilizing more eco-friendly materials in the aforementioned applications. CSP is derived from renewable resources and provides several benefits, including availability, abundance, and low cost. Additionally, CSP features high porosity, a specific surface area, it is light weight, and it has been proven to have remarkable moisture adsorption capacity, capable of transporting moisture from the surface to the interior [16,17]. At present, the practical applications of modifying the chemical composition and physical structure of CSP using green SE technology mainly focus on the fields of biofuels and pulp production [27,28]. It is worth noting that our team was the first to modify CSP using this technology to develop a new type of super-hygroscopic material (SE-CSP), which is highly innovative. Then, by investigating the chemical components, micro-morphology, porosity, physical structure, surface properties, chemical functional groups, and elemental composition of SE-CSP, we conducted a detailed analysis of its hygroscopic properties, including adsorption kinetics, isotherms, and thermodynamics, to elucidate the underlying adsorption mechanism.

## 2. Materials and Methods

### 2.1. Materials

Corn straw was sourced from Xiangyu Co., Ltd. (Changchun, China). The CSP was manually removed and cut into sections shorter than 20 mm.

### 2.2. Preparation of SE-CSP

The SE-CSP was prepared following the method reported by Bu et al. [29]. The dried CSP samples were placed in the material bin of the SE machine (QB-300, Simingte Co., Ltd., Jinan, China). Each time, 80 g of material was blasted. The hatch cover was then tightened, the blasting pressure was set at 1.0 MPa, and the temperature was 184 °C. When the pressure inside the explosive body reached the preset pressure, the steam valve was opened. After maintaining the pressure for 90 s, the steam valve was quickly closed to carry out the explosion. The treated materials, referred to as the SE-CSP samples, were gathered and dried at 40 °C in preparation for testing.

### 2.3. Carbohydrate Analysis

The composition of cellulose, hemicellulose, and lignin in the samples was analyzed following the procedure outlined by the National Renewable Energy Laboratory (NREL) for assessing structural carbohydrates and lignin within biomass. For the acid hydrolysis of the samples, sulfuric acid (72%, *w*/*w*) was employed and maintained at 30 °C for 1 h in a water bath. Following this, the hydrolyzed samples were diluted to achieve a 4% acid concentration and subsequently placed in an autoclave at 121 °C for 1 h. The samples were then vacuum-filtered using a filtration crucible. The crucible holding the acid-insoluble residue (AIR) was dried until it reached a constant weight. Subsequently, it was transferred to a muffle furnace and maintained for ashing, enabling the measurement of the acid-insoluble lignin (AIL) content. The content of acid-soluble lignin (ASL) in the hydrolyzate was determined using a UV-visible spectrophotometer (UV-1800, Shimadzu, Tokyo, Japan) at 280 nm. The overall lignin content was represented as the total of AIL and ASL combined. Monosaccharides in the hydrolysate were identified and quantified through High-Performance Liquid Chromatography (HPLC) (Agilent 1260 series, Agilent Technologies, Santa Clara, CA, USA). The cellulose content was quantified based on glucose equivalence, while the hemicellulose level was determined by the cumulative equivalence of xylose, arabinose, galactose, and mannose found in the hydrolysis solution.

### 2.4. Structural Characterization

#### 2.4.1. Confocal Laser Scanning Microscopy (CLSM)

Thin transverse sections, measuring 35 μm in thickness, were obtained from the samples using a JYD215 rotary microtome (Lai Boruijie Technology Co., Ltd., Beijing, China). Prior to CLSM analysis, the cross sections were stained with 0.1% aqueous safranin O for 5 min. Following this, the sections were placed in immersion oil for microscopic examination. As previously reported [30,31], imaging was performed using the Zeiss LSM 700 confocal microscope (Carl Zeiss AG, Oberkochen, Germany). A 488 nm laser was employed for excitation, and fluorescence emission was detected via a long-pass 590 nm filter.

#### 2.4.2. Scanning Electron Microscopy (SEM)

A SEM (SU9000, Hitachi, Tokyo, Japan) was utilized to examine the morphology of the samples. The samples were fixed on double-sided tape and observed after being sputtered with a gold layer.

#### 2.4.3. Specific Surface Area and Pore Volume Analysis

The adsorption-related characteristics were determined using N_2_-adsorption/desorption isotherms with an automated specific surface area analyzer (ASAP 2020M, Micromeritics Instruments, Norcross, GA, USA) at −196 °C. The N_2_ adsorption data were analyzed based on the BET model to determine the specific surface area [32]. The pore volume was determined automatically using the Barrett–Joyner–Halenda model [33].

#### 2.4.4. Fourier Transform Infrared Spectroscopy (FTIR)

The chemical transformations in the samples were examined using an FTIR device (Frontier NIR-MID spectrometer, PerkinElmer Inc., Waltham, MA, USA) within the range of 400–4000 cm^−1^ [34].

#### 2.4.5. X-Ray Photoelectron Spectroscopy (XPS)

The XPS spectra of the samples were acquired using an XPS spectrometer (Kalpha, Thermo, Waltham, MA, USA) equipped with an Al anode. Surface composition surveys were measured with an energy step size of 1.0 eV and a pass energy of 100 eV. High-resolution spectra of the O 1s, N 1s, and C 1s regions were obtained with an energy step size set at 0.05 eV and a pass energy configured at 50 eV. The atomic ratio of oxygen-to-carbon (O/C) was determined based on their normalized peak areas as shown in Equation (1) [35]:

(1) $$\frac{O}{C}=\frac{I_{O}}{I_{C}}\times \frac{S_{C}}{S_{O}}$$

where *I_O_* and *I_C_* represent the standardized integral regions of the oxygen and carbon peaks, respectively, while *S_C_/S_O_* serves as the corrected term for the sensitivity factor.

#### 2.4.6. X-Ray Diffraction (XRD)

The crystalline structures of the samples were analyzed using XRD (X’Pert3 Powder 10300, Panalytical, Almelo, The Netherlands) in the 2θ range from 5° to 80° [36].

#### 2.4.7. Thermogravimetric Analysis (TGA)

The thermal stability of the samples was evaluated using a TGA 8000 instrument (PerkinElmer Management Co., Shanghai, China) in the range of 50 to 600 °C.

#### 2.4.8. Contact Angle

The static water contact angles of the samples were determined using a contact angle analyzer (JY-82B Kruss DSA, Dataphysics, Filderstadt, Germany). The powder of the samples was compressed into thin, circular flakes. A droplet of distilled water (5 μL) was gently deposited onto the surface of these flakes using an automatic pipette. Once the water droplet settled and stabilized on the sample surface, images were promptly recorded.

### 2.5. Moisture Adsorption and Retention Experiment

Moisture adsorption characteristics of the samples were examined using a Dynamic Vapor Sorption (DVS) instrument (DVS Advantage, Surface Measurement Systems, Middlesex, UK). Mass variations in the samples were recorded between 25 and 35 °C and across a relative humidity (RH) range of 0–98%. The starting weights of the dried samples were noted as *W* (g), while their weights were periodically assessed and documented as *W_n_* (g). The equilibrium moisture adsorption (%) was determined using Equation (2) [37]:

(2) $$Moisture\;adsorption(\%)=\frac{W_{n}-W}{W}\times 100$$

The dried samples were initially hydrated using deionized water and subsequently weighed, with the original mass being denoted as *H* (g). Afterward, the samples were transferred to a desiccator. Their weights were periodically measured and recorded as *H_n_* (g). The moisture retention (%) was determined using Equation (3) [37]:

(3) $$Moisture\;retention(\%)=\frac{H-H_{n}}{H}\times 100$$

### 2.6. Moisture Adsorption Model Fitting

To understand the moisture adsorption mechanism of the samples, isothermal moisture adsorption experiments and kinetic model fitting were performed [37,38,39]. The equations for the moisture adsorption models are shown in Table 1.

### 2.7. Statistical Analysis

All experiments were conducted three times, and the results are presented as the mean ± standard deviation. Statistical analysis was carried out using SPSS Statistics software (Version 21.0, SPSS Inc., Chicago, IL, USA). The significance of differences was evaluated by Duncan’s multiple range test and one-way ANOVA, with a threshold of *p* < 0.05.

## 3. Results

### 3.1. Composition Analysis

The chemical composition results showed (Table 2) that the content of cellulose, hemicellulose, and lignin in the raw CSP was 61.55%, 26.04%, and 12.41% (*w*/*w*), respectively. In contrast, the cellulose content in SE-CSP significantly increased to 79.45% (*w*/*w*) (*p* < 0.05), whereas the hemicellulose and lignin levels significantly decreased to 15.42% and 5.13% (*w*/*w*), respectively (*p* < 0.05). The reason may be that SE treatment reduced the hemicellulose content by loosening the bonds between lignin and hemicellulose [40]. The degradation of lignin was caused by the severing of β-O-4 and β-5 aryl ether bonds in its structure due to SE treatment [41]. Furthermore, the high-pressure steam treatment led to significant solubilization of hemicellulose and lignin as a result of the direct interaction between these components and the steam [42]. Upon the removal of lignin and hemicellulose, the cell wall layers underwent partial exfoliation, which led to the formation of pores and voids within the modified CSP, thereby improving its adsorption properties.

### 3.2. CLSM Analysis

**Figure 1:**
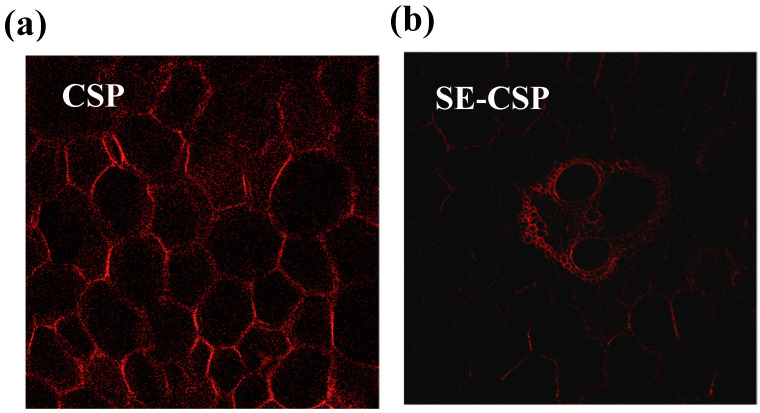
Confocal laser scanning microscopy (CLSM) images of the lignin distribution of CSP before and after SE treatment: (**a**) CSP; (**b**) SE-CSP.

The lignin present in the cell walls of straw acts as a natural adhesive, covalently linking hemicellulose molecules. Studies have shown that removing lignin from the cell walls is an effective means of enhancing the adsorptive ability of straw [43]. Lignin has fluorescent properties. The intrinsic fluorescence of lignin itself was observed to be green by CLSM. In order to observe more clearly the distribution and content changes in lignin in CSP before and after SE treatment, in this study, we stained it with 0.1% aqueous safranin O. Aqueous safranin O is a dye with a positive charge. Lignin molecules contain a large number of hydroxyl and carboxyl groups, and these groups can carry a negative charge. These negatively charged groups in lignin can combine with the positively charged safranin O through ionic bonds or hydrogen bonds, thereby achieving dyeing. It is worth noting that aqueous safranin O has a strong affinity for lignin, but has no dyeing effect on cellulose and hemicellulose, which precisely meets our dyeing requirements. The research by Kang et al. [44] also utilized the staining of lignin in hybrid Pennisetum with aqueous safranin O and then observed its distribution and content changes through CLSM. Therefore, in this study, the lignin in CSP was stained with 0.1% aqueous safranin O, and then the distribution and content changes before and after SE treatment were observed by CLSM. Fluorescence mapping localized lignin distribution based on the intensity of fluorescence, which exhibited a linear relationship with the lignin content [35]. As shown in Figure 1, the red fluorescence signal indicated the distribution and content of lignin. In raw CSP, the fluorescence signal of lignin was primarily present in the cell middle lamella, cell corner, and secondary cell walls (Figure 1a). In the SE-CSP sample, the dense network of the lignin structure was disrupted, as indicated by the attenuated lignin fluorescence intensity (Figure 1b). The reason may be that high temperatures and high pressures during SE treatment effectively removed some of the lignin and hemicellulose, thus loosening the material structure, which contributed to enhancing its sorption performance [21].

### 3.3. Morphology Analysis

**Figure 2:**
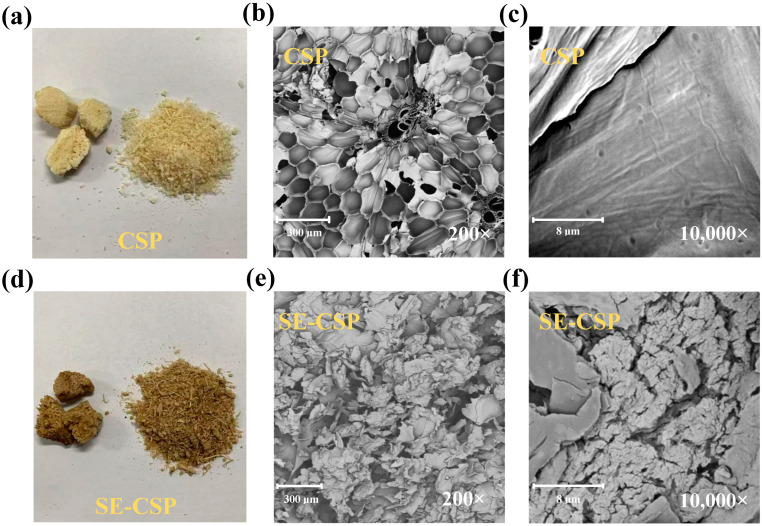
Photographs and scanning electron microscopy (SEM) images of CSP before and after SE treatment: (**a**–**c**) CSP; (**d**–**f**) SE-CSP.

Raw CSP exhibited a honeycomb-like cellular architecture characterized by thin cell walls and an overall dense structure (Figure 2a). After the SE treatment, the structure of the material became looser and changed from its original light-yellow color to dark brown (Figure 2d), which was attributed to the high temperature and presence of water that led to partial lignin degradation and exposed numerous light-absorbing chromophoric groups [45]. SEM images showed that SE-CSP (Figure 2e,f) had an interwoven tubular-lamellar structure, and the overall structure was looser and more expansive than that of CSP (Figure 2b,c). It featured an irregular surface with numerous pores and folds, as well as exposed internal fibers. One potential explanation for this phenomenon is that, during the SE processing, the material experienced high temperatures and pressures. These conditions led to the evaporation of internal moisture and a dramatic expansion in its volume, which in turn caused the surface and internal fibers to break and form pores. As a result, the originally dense structure was disrupted [29]. These structural features conferred SE-CSP with a higher specific surface area and porosity, while allowing more hydrophilic groups and other water-binding sites to be exposed on the fiber surface, which facilitated water vapor penetration into its internal structure, thereby enhancing both the hygroscopic property and rate [37]. Similar observations were also reported in previous studies on SE-modified rice husk [46] and bamboo [47], indicating that SE facilitated the depolymerization of the internal structure and altered the surface morphology of these materials, consequently enhancing their adsorption properties.

### 3.4. Specific Surface Area and Pore Volume Analysis

Compared with CSP, SE-CSP demonstrated a considerably greater specific surface area, which increased from 7.69 to 33.96 m^2^/g, along with a larger pore volume, rising from 0.03 to 0.05 cm^3^/g (Table 3). This enhancement was ascribed to the adiabatic expansion and bursting of water within the CSP when the SE pressure was released. This phenomenon caused the macromolecular fibers to break and made the internal structure less compact [48]. Consequently, this resulted in an increase in both the pore size and volume of the material, as well as a greater number of wrinkles and pores, thereby enhancing the specific surface area [49]. As a result, more space was provided for water molecule accommodation, which contributed to improving the hygroscopic property of the material.

### 3.5. FTIR Analysis

**Figure 3:**
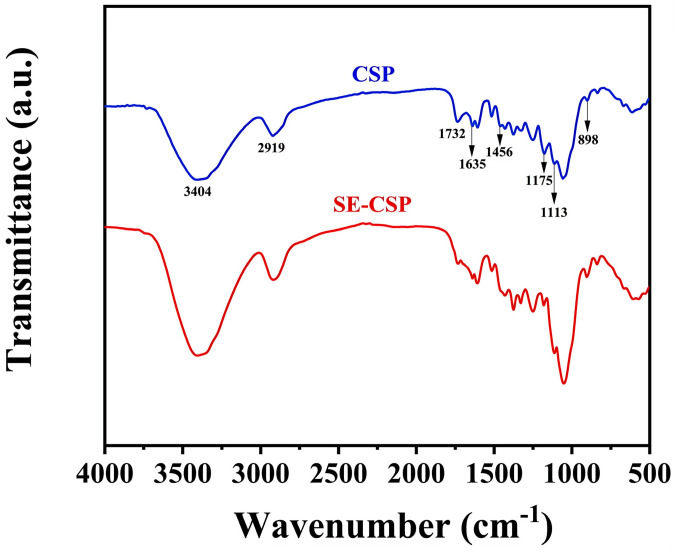
Fourier transform infrared spectroscopy (FTIR) spectra of CSP before and after SE treatment.

Figure 3 illustrates the transformations of chemical bonds and functional groups within the samples. The strong similarity between the spectra of CSP and SE-CSP indicates that no new functional groups or chemical bonds formed after SE treatment, but rather only changes in the chemical composition content occurred. The absorption band at 3404 cm^−1^ (within a range of 3200 to 3650 cm^−1^), attributed to the stretching vibration of the hydroxyl group in cellulose and hemicellulose, is influenced by inter- and intramolecular hydrogen bonding. Compared with the absorption band intensity of CSP, that of SE-CSP was higher, suggesting a greater exposure of hydroxyl groups and enhanced inter- and intramolecular interactions between the polysaccharide chains, which contributed to its hydrophilic properties [50]. The absorption band at 2919 cm^−1^ (within a range of 2500 to 3200 cm^−1^) can be attributed to the stretching vibration of methyl and methylene C–H in cellulose, while the bands at 1113 cm^−1^ (within a range of 1090 to 1140 cm^−1^) and 898 cm^−1^ (within a range of 850 to 950 cm^−1^) are caused by the ring vibration of the glycosidic bonds, which are designated as characteristic bands of the cellulose structure [21]. The intensities of these characteristic absorption bands of SE-CSP were enhanced to varying degrees compared to those of CSP, indicating an increase in cellulose content. This occurred because the SE treatment disrupted the tight cross-linking structure among cellulose, hemicellulose, and lignin, thus exposing more cellulose and enhancing its characteristic absorption bands [51]. The band at 1732 cm^−1^ (within a range of 1680 to 1800 cm^−1^) represented the C=O stretching vibration related to hemicellulose, which almost disappeared in the SE-CSP spectra. Hemicellulose was the primary chemical component of the materials, indicating that it had been highly degraded during the SE process. The same phenomenon occurred in the hemicellulose correlation absorption bands at 1175 cm^−1^ (within a range of 1140 to 1200 cm^−1^) (C–O–C stretching vibration) [52]. The band at 1635 cm^−1^ (within a range of 1610 to 1680 cm^−1^) was associated with the aromatic skeletal vibration of lignin, while the band at 1456 cm^−1^ (within a range of 1435 to 1490 cm^−1^) represented C-H deformation combined with vibrations of aromatic rings. The two absorption band intensities of SE-CSP were lower than those of CSP, indicating that SE treatment had excellent lignin removal capability [23]. Briefly, the SE modification treatment did not introduce new chemical groups into the material but altered the extent of exposed chemical groups and modified the content of chemical compositions. These changes contributed to enhancing the hygroscopic capacity of the material [43].

### 3.6. XPS Analysis

**Figure 4:**
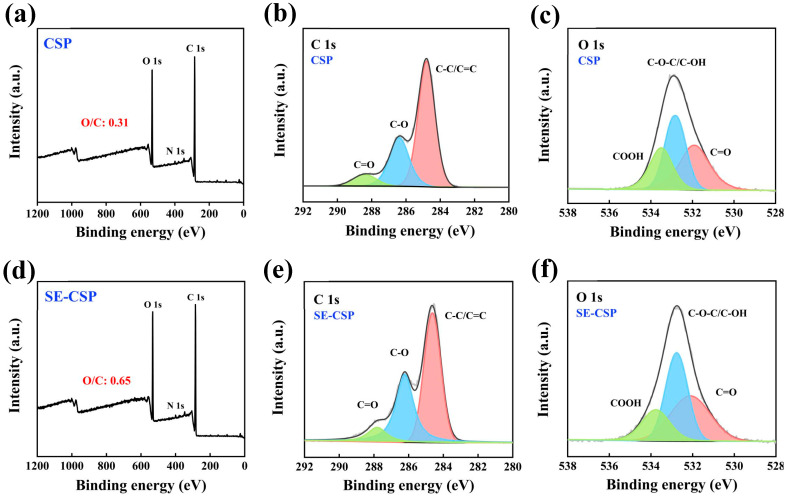
X-ray photoelectron spectroscopy (XPS) wide-scan spectra of CSP before and after SE treatment: (**a**) CSP; (**d**) SE-CSP. The C 1s and O 1s high-resolution XPS spectra for CSP before and after SE treatment: (**b**,**c**) CSP; (**e**,**f**) SE-CSP.

The samples’ surface functional groups and elemental composition were further examined through XPS analysis. As shown in Figure 4a,d, the XPS full-spectrum scan of CSP and SE-CSP revealed three distinct peaks at approximately 285.03 eV (C 1s), 400.00 eV (N 1s), and 532.01 eV (O 1s). The straw materials essentially consist of cellulose, hemicellulose, and lignin, which have known theoretical O/C atomic ratios of 0.83, 0.81, and 0.33, respectively [53]. The O/C atomic ratio in CSP was determined to be 0.31, closely aligning with the previously documented value of 0.33 [54]. After SE treatment, the O/C atomic ratio of SE-CSP was increased to 0.65. This may be attributed to the SE treatment decreasing the polymerization degree of cellulose in the material, exposing more hydroxyl groups on the surface, and thus increasing the O/C ratio [35]. Moreover, SE treatment destroyed the tight network structure of cellulose–hemicellulose–lignin, thereby enhancing the cellulose surface accessibility and consequently leading to an increase in the O/C ratio [55]. Figure 4b,c,e,f show the high-resolution spectra of C 1s and O 1s for CSP and SE-CSP. The peak assignments and relative content of functional groups are shown in Table 4. The C1s spectrum of the samples was deconvoluted into three components. The peak at 284.71 eV corresponded to C–C/C=C groups, while the peaks at 286.40 and 288.41 eV were indicative of C–O and C=O groups [56]. The O1s spectrum was deconvoluted into three peak components. Specifically, the peaks at 531.92, 532.90, and 533.50 eV were attributed to C=O, C−O−C/C−OH, and COOH groups, respectively [47]. Chundawat et al. [57] reported that in fibrous materials, the biomass components contribute to multiple emission peaks of carbon atoms as follows: cellulose contributes approximately 85% of its signal to C–O peaks and 15% to C=O peaks; hemicellulose contributes about 80% of its signal to C–O peaks and 20% to C=O peaks; lignin contributes 50% of its signal to C–C/C=C peaks and 50% to C–O peaks. The content of the C–C/C=C, C–O, and C=O groups in CSP was 62.72%, 29.32%, and 7.96%, respectively. After SE treatment, the content of the C–C/C=C groups significantly decreased to 51.84% (*p* < 0.05), while the content of the C–O and C=O groups significantly increased to 39.10% and 9.06% (*p* < 0.05), respectively. This could be attributed to the SE treatment, which led to a reduction in lignin and hemicellulose content while simultaneously increasing the cellulose content of the material. Taking into account the FTIR and XPS results, SE clearly affected both the non-cellulosic and cellulosic components of the straw, consistent with previously published findings [58,59]. In general, SE treatment decreased the degree of polymerization of cellulose in the material, thereby exposing more hydroxyl groups and enhancing its hydrophilic properties. Additionally, SE treatment effectively removed the non-fiber components (e.g., hemicellulose and lignin) from the material, leading to a loosened internal structure that further increased its hygroscopic properties.

### 3.7. XRD Analysis

**Figure 5:**
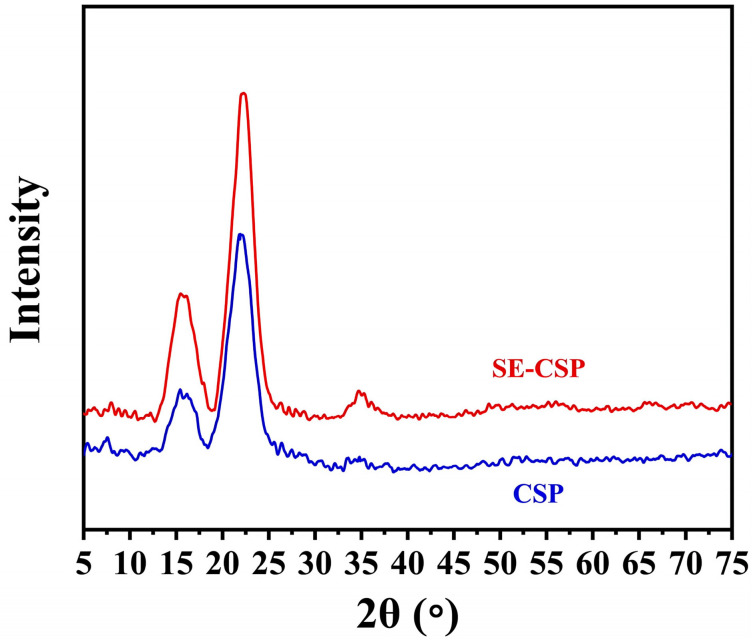
X-ray diffraction (XRD) diffraction pattern of CSP before and after SE treatment.

The raw CSP exhibited two weak peaks at 15.51° and 34.70°, and one strong peak at 22.20° (Figure 5). These were ascribed to cellulose I crystallites, corresponding to the crystalline planes (110), (004), and (200), respectively. The diffraction pattern of SE-CSP exhibited peaks that closely resembled those observed in CSP, suggesting that the SE treatment had no significant impact on the crystal structure, consistent with previously published findings [60]. The relative crystallinity index (CrI) of SE-CSP increased to 29.66%, compared with 25.47% for CSP. This increase in CrI may have occurred because, in contrast to the crystalline regions formed by cellulose, the amorphous regions composed of hemicellulose and lignin were more susceptible to destruction by the SE treatment, leading to a relative increase in the crystalline regions [61]. Sun et al. [58] found that the crystallinity, porosity, and surface area of the straw fiber materials increased due to the ordered rearrangement and secondary crystallization occurring in the non-crystallized and imperfect parts of the cellulose crystal structure. This allowed more water molecules to ingress into the material’s internal structure, thereby increasing its hygroscopic property.

### 3.8. TGA

**Figure 6:**
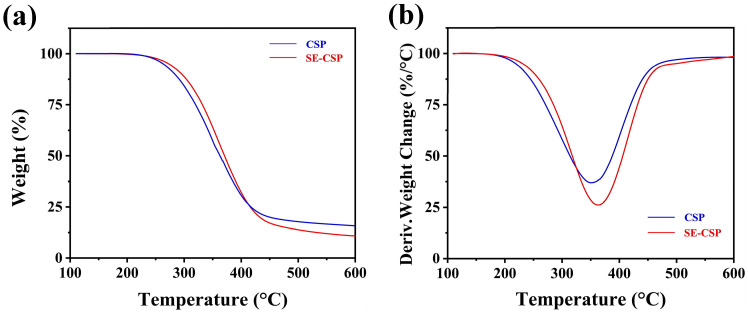
(**a**) Thermogravimetric (TG) and (**b**) derivative thermogravimetric (DTG) curves of CSP before and after SE treatment.

Generally, four transitional states are identified in fibrous materials: moisture evaporation, hemicellulose breakdown, cellulose decomposition, and lignin degradation. As shown in the TG curve (Figure 6a), no weight loss related to water evaporation was observed around 100 °C. This observation was because both CSP and SE-CSP samples were stabilized at 110 °C for 10 min before measurements. Hemicellulose began to degrade between 200 and 300 °C, followed by cellulose degradation from 275 to 450 °C [35,62]. Lastly, lignin decomposed over a wider temperature region from 450 to 700 °C due to its complex structure [48]. Notably, SE-CSP exhibited a significantly higher decomposition temperature compared to CSP, indicating improved thermal stability. As shown in the derivative thermogravimetric (DTG) curve (Figure 6b), the onset degradation temperatures of CSP and SE-CSP were 262.50 and 287.51 °C, respectively. The improved degradation temperature was attributed to the thermal depolymerization of hemicelluloses and pectin during SE treatment [50]. The DTG peak temperature for CSP was 350.15 °C, while the SE-CSP DTG peak shifted to a higher temperature of 362.53 °C, which was associated with significant cellulose decomposition. The SE-CSP DTG peak exhibited greater intensity compared to that of CSP, suggesting a potentially higher cellulose content [63]. Furthermore, studies have indicated that fibrous materials with greater lignin content produce a higher char yield when subjected to elevated temperatures. At 600 °C, CSP yielded a carbon residue of 16.23%, whereas SE-CSP had a char content of approximately 11.06%, indicating that SE treatment effectively facilitated the removal of lignin. These results suggested that the pyrolysis temperature and thermal stability of the materials were enhanced after SE treatment due to the partial removal of hemicellulose and lignin and the higher cellulose crystallinity, which facilitated further functionalization and practical application of the material [64].

### 3.9. Surface Wettability Analysis

**Figure 7:**
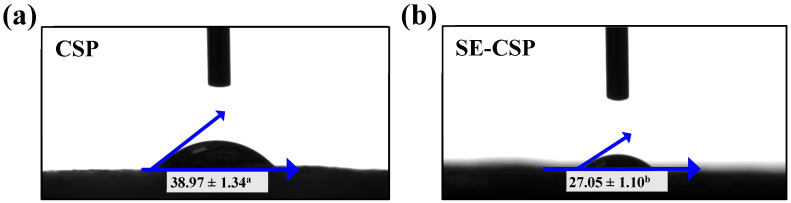
Static water contact angle images of CSP before and after SE treatment: (**a**) CSP; (**b**) SE-CSP. Different lowercase letters within a line indicate significant differences in values (*p* < 0.05).

Figure 7a,b show that, compared to raw CSP, the contact angle of SE-CSP significantly decreased from 38.97° to 27.05° (*p* < 0.05), indicating greater hydrophilicity. One reason for this is that SE treatment removed the non-cellulosic hydrophobic surface layer of the material, exposed more hydrophilic cellulose, and increased the concentration of polar oxygen-containing groups, thereby enhancing its hydrophilicity [22,55]. Another possible reason is that the lignin in the material was degraded by SE treatment, thereby increasing the accessibility of water molecules. Studies have demonstrated that native lignin exhibits greater hydrophobicity compared to the more hydrophilic cellulose [35]. Additionally, the structural features of SE-CSP with a higher specific surface area and porosity could promote water molecule transport from the surface to the interior, thus enhancing the surface wettability of the material and laying the foundation for its outstanding moisture adsorption and retention capacity [65].

### 3.10. Moisture Adsorption and Retention Analysis

**Figure 8:**
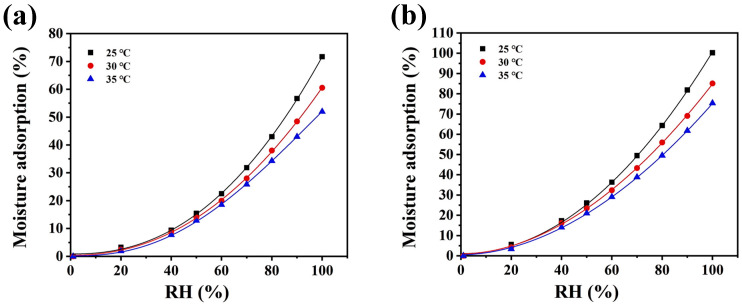
Experimental data and adsorption isotherms of the Guggenheim–Anderson–de Boer (GAB) model of CSP before and after SE treatment: (**a**) CSP; (**b**) SE-CSP.

Figure 8a,b indicate that, based on the classification by the International Union of Pure and Applied Chemistry, the isothermal moisture adsorption curves of CSP and SE-CSP correspond to type III [66]. The adsorption isotherms of the two materials showed the same trend, with their equilibrium moisture adsorption percentage gradually decreasing as the temperature increased, indicating an exothermic process [67]. This phenomenon occurred because the elevated temperature increased the activation energy of water molecules and weakened their binding force with the material surface. Consequently, the water molecules were able to break free from the binding site, resulting in a reduction in the equilibrium moisture adsorption percentage [38]. Under identical conditions, SE-CSP exhibited higher equilibrium moisture adsorption than CSP, indicating that SE treatment effectively enhanced the material’s hygroscopic properties. Previous studies have confirmed that the hygroscopic properties of materials depend on their microstructure, chemical composition, and specific surface area [68]. Therefore, the results here may be attributed to the SE treatment disrupting the inter-fiber connecting bonds in the material, altering its surface structure and exposing more hydrophilic groups and other water-binding sites, ultimately increasing the material’s affinity for water molecules [50]. Moreover, high temperature and high pressure caused by SE treatment increased the exfoliation of the cell wall layers, promoting lignin and hemicellulose removal, which effectively enhanced material permeability and facilitated water molecule diffusion and adsorption within the internal voids, ultimately improving the material’s hygroscopic properties [23].

The isothermal moisture adsorption data were fitted using the GAB, Oswin, Smith, and Peleg models, and the corresponding parameters are shown in Table 5. The GAB and Peleg models had high determination coefficients (R^2^ > 0.99) and low residual sum of squares (RSS), indicating excellent model fit and predictive accuracy [69]. However, the Peleg model is an empirical mathematical model that cannot fully reveal the hygroscopic properties of the samples; therefore, the GAB model was employed to better characterize these properties [39]. As shown in Figure 8a,b, the GAB model revealed that the hygroscopic processes of both CSP and SE-CSP followed the law of multi-layer adsorption [38]. Hydrophilic groups on the surfaces of the materials acted as active adsorption sites in a humid environment and directly formed strong intermolecular forces with water molecules, producing single-layer moisture adsorption on the surface. As the coverage of water molecules on the material’s surface increased, intermolecular interactions formed between water molecules and other active groups, which gradually penetrated the interior of the materials via their pores during multi-layer moisture adsorption [70].

The RSS was calculated using Equation (4) [71]:

(4) $$RSS={\sum_{i=1}^{n}\left|Y_{i}-{\tilde{Y}}_{i}\right|}^{2}$$

where *n* is the number of data points, *Y_i_* is the actual value, and *Ỹ_i_* is the predicted value.

The thermodynamic parameters were calculated from the equilibrium moisture adsorption data at 80% RH and various temperatures using the van ’t Hoff equations [72], as follows:

(5) $$Ln(\frac{q_{e}}{RH})=LnK_{d}=\frac{\Delta S}{R}-\frac{\Delta H}{RT}$$

(6) ΔG=ΔH−T×ΔS

where *K_d_* is the equilibrium distribution coefficient, *q_e_* (mg/g) is the equilibrium moisture adsorption, *T* (K) is the temperature, *R* (8.314 J/mol·K) is the ideal gas constant, and Δ*H* (kJ/mol), Δ*S* (kJ/mol·K), and Δ*G* (kJ/mol) are the enthalpy change, entropy change, and Gibbs free energy, respectively.

As shown in Table 6, Δ*G* < 0 and Δ*H* < 0, indicating that the hygroscopic process was spontaneous, enthalpy-driven, and exothermic in nature [73,74]. Furthermore, the absolute value of Δ*G* (|Δ*G*|) decreased as temperature increased, implying that lower temperatures were more favorable for water molecule adsorption. Δ*S* < 0 indicated a reduction in molecule disorder at the solid/liquid interface [75]. Generally, the absolute value of Δ*H* (|Δ*H*|) for physical adsorption is typically less than 20 kJ/mol, while that for chemisorption ranges from 80 to 200 kJ/mol [76]. The |Δ*H*| for both CSP and SE-CSP was below 20 kJ/mol, suggesting that the adsorption processes of water molecules on both materials should be regarded as physical adsorption [77]. It was inferred that the hygroscopic process of CSP and SE-CSP involved water molecules initially entering the material surfaces via physical adsorption, subsequently binding to hydrophilic groups and other water-binding sites through hydrogen bonds and van der Waals forces, and ultimately being retained within the materials as bound water [38]. Furthermore, compared with CSP, SE-CSP had a higher |Δ*H*| value, which indicated that SE-CSP had a stronger affinity with water molecules and exhibited more excellent moisture adsorption performance [78].

**Figure 9:**
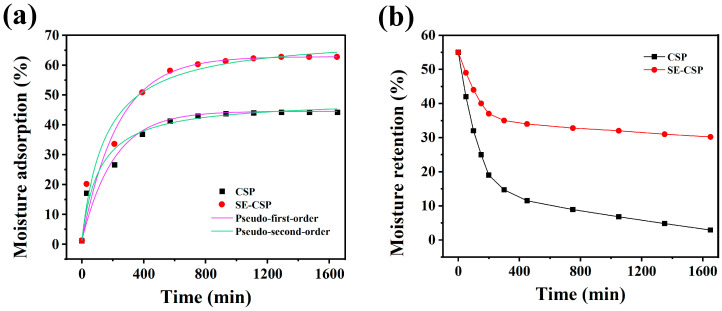
(**a**) Adsorption kinetics and (**b**) moisture retention of CSP before and after SE treatment.

The adsorption kinetic data and fitting curves for the samples are shown in Figure 9a. Both CSP and SE-CSP absorbed moisture quickly at 80% RH and 25 °C, and the moisture adsorption percentage of SE-CSP increased rapidly. The percent moisture adsorption of SE-CSP (62.50%) was higher than that of CSP (44.01%) after 1650 min, indicating that SE-CSP had more adsorption sites and a greater moisture adsorption capacity. The parameters of the fitted models are shown in Table 7. Based on the determination coefficient (R^2^), the hygroscopic kinetic models of CSP and SE-CSP were found to conform more closely to the pseudo-first-order kinetic model. The predicted values of the saturated moisture adsorption percentage for CSP and SE-CSP were 45.00% and 62.67%, respectively, showing close agreement with the experimental data. These results indicated that the moisture adsorption processes of both CSP and SE-CSP were physical adsorption, consistent with previous conclusions [79].

The moisture retention effects of the samples are shown in Figure 9b. The dehydration rate of SE-CSP was significantly slower compared to that of CSP. After 1650 min under dry conditions, CSP had nearly achieved complete desiccation, while SE-CSP retained 30.22% of its moisture. This was likely due to SE-CSP having more hydrophilic groups, which enhanced binding with water molecules [70]. Meanwhile, the extensive internal network structure also provided binding sites for water molecules, making it more difficult for water molecules to detach from the material [37]. Overall, the SE-CSP obtained by SE-modification treatment exhibited excellent moisture adsorption and retention properties, making it suitable as a novel super-hygroscopic material.

## 4. Conclusions

The present study used CSP as a raw material to prepare a novel super-hygroscopic material (SE-CSP) using a green and efficient SE modification method. The results confirmed that SE effectively removed lignin and hemicellulose, disrupted the thin cell walls of natural CSP, and formed an aligned porous structure with capillary channels. SE changed the bonding distribution and surface morphology and enhanced the crystallinity and thermal stability of CSP. Notably, the changes in structure and chemical composition of CSP induced by SE treatment resulted in an increase in both the pore volume and specific surface area of the material, as well as the exposure of more hydrophilic groups and other water-binding sites, which contributed to enhancing its hygroscopic properties. The equilibrium hygroscopic percentage of SE-CSP (62.50%) was higher than that of CSP (44.01%) at 25 °C and 80% RH, indicating significantly greater hygroscopicity. The hygroscopic process of SE-CSP followed a Type III isotherm and fitted GAB, Peleg, and pseudo-first-order kinetic models. This process exhibited multi-layer adsorption with enthalpy-driven, exothermic behavior, primarily through physical adsorption involving hydrogen bonds and van der Waals forces. These findings indicated the feasibility of SE-CSP as a novel super-hygroscopic material and also provided useful information for the high value-added and sustainable development of other fiber-rich agricultural wastes.

## Figures and Tables

**Table 1:** Moisture adsorption models.

Model	Model Equations
GAB	$W=\frac{C\times K\times m_{0}\times RH}{\left(1-K\times RH)\times (1+(C-1)\times K\times RH\right)}$
Oswin	$W=A\times {\left(\frac{RH}{1-RH}\right)}^{B}$
Smith	W=P+Q×ln(1−RH)
Peleg	$W=E_{1}\times RH^{n_{1}}+E_{2}\times RH^{n_{2}}$
Pseudo-first-order	$\ln(q_{e}-q_{t})=\ln q_{e}-k_{1}\times q_{t}$
Pseudo-second-order	$\frac{t}{q_{t}}=\frac{1}{k_{2}\times {q_{e}}^{2}}+\frac{t}{q_{e}}$

Note: *W* (% d.b.) is the equilibrium moisture content; *m*_0_ (% d.b.) is the monolayer equilibrium moisture content; *K*, *C*, *A*, *B*, *P*, *Q*, *E*_1_, *E*_2_, *n*_1_, *n*_2_, *k*_1_, and *k*_2_ are model constants; *t* is the moisture adsorption time; *q_e_* (%) is the model-predicted value of the saturated moisture content; *q_t_* (%) is the equilibrium moisture content at time *t*.

**Table 2:** Composition analysis of CSP before and after SE treatment.

Total Dry Weight (% *w*/*w*)		
	CSP	SE-CSP
Cellulose	61.55 ± 1.55 ^a^	79.45 ± 2.23 ^b^
Hemicellulose	26.04 ± 1.86 ^a^	15.42 ± 2.03 ^b^
Lignin	12.41 ± 1.15 ^a^	5.13 ± 0.93 ^b^

Note: Different lowercase letters within a line indicate significant differences in values (*p* < 0.05).

**Table 3:** Specific surface area and pore volume of CSP before and after SE treatment.

Parameter	CSP	SE-CSP
Specific surface area (m^2^/g)	7.69 ± 1.07 ^a^	33.96 ± 1.84 ^b^
Pore volume (cm^3^/g)	0.03 ± 0.01 ^a^	0.05 ± 0.01 ^b^

Note: Different lowercase letters within a line indicate significant differences in values (*p* < 0.05).

**Table 4:** The relative content of surface functional groups in the C1s and O1s spectra of CSP before and after SE treatment.

Region	Functional Group	CSP		SE-CSP	
		Binding Energy (eV)	Relative Content (%)	Binding Energy (eV)	Relative Content (%)
C 1s	C–C/C=C	284.71	62.72 ± 2.21 ^a^	284.71	51.84 ± 1.58 ^b^
	C–O	286.40	29.32 ± 1.96 ^a^	286.40	39.10 ± 1.24 ^b^
	C=O	288.41	7.96 ± 0.75 ^a^	288.41	9.06 ± 0.69 ^b^
O 1s	C=O	531.92	35.63 ± 2.55 ^a^	531.92	38.09 ± 2.02 ^b^
	C−O−C/C−OH	532.90	39.52 ± 1.43 ^a^	532.90	41.72 ± 2.01 ^b^
	COOH	533.50	24.85 ± 1.02 ^a^	533.50	20.19 ± 0.96 ^b^

Note: Different lowercase letters within a line indicate significant differences in values (*p* < 0.05).

**Table 5:** Evaluation parameters of the different adsorption isotherm models of CSP before and after SE treatment.

Model	Samples	CSP			SE-CSP		
	T/°C	25	30	35	25	30	35
GAB	R^2^	0.9996	0.9994	0.9997	0.9997	0.9992	0.9997
	Residual sum of squares (RSS)	0.72465	0.80924	0.43278	0.45688	1.25312	0.41499
Oswin	R^2^	0.9233	0.9478	0.9150	0.9378	0.9413	0.9407
	RSS	86.8218	57.8938	105.3512	70.4709	54.0246	50.2566
Smith	R^2^	0.9764	0.9823	0.9503	0.9645	0.9733	0.9780
	RSS	42.3156	30.0099	73.4073	60.1222	44.9087	42.8163
Peleg	R^2^	0.9998	0.9997	0.9992	0.9995	0.9998	0.9999
	RSS	0.2342	0.2710	0.3896	0.3044	0.2367	0.2103

**Table 6:** Thermodynamic parameters of moisture adsorption of CSP before and after SE treatment.

Samples	Δ*S* (kJ/mol·K)	Δ*H* (kJ/mol)	Δ*G* (kJ/mol)		
			298 K	303 K	308 K
CSP	−0.042	−16.81	−4.29	−4.08	−3.87
SE-CSP	−0.048	−19.32	−5.02	−4.78	−4.54

**Table 7:** Evaluation of different adsorption kinetic models of CSP before and after SE treatment.

Samples	q_e_-exp (%)	Pseudo-First-Order			Pseudo-Second-Order		
		q_e_ (%)	k_1_ (1/min)	R^2^	q_e_ (%)	k_2_ (g/mg·min)	R^2^
CSP	44.01	45.00	0.0047	0.9844	45.14	0.0002	0.9321
SE-CSP	62.50	62.67	0.0045	0.9723	64.21	0.0001	0.9506

Note: q_e_-exp is the experimental value of equilibrium moisture adsorption.

## Data Availability

The original contributions presented in this study are included in the article. Further inquiries can be directed to the corresponding author.
